# Non-beneficial admission to the intensive care unit: A nationwide survey of practices

**DOI:** 10.1371/journal.pone.0279939

**Published:** 2023-02-02

**Authors:** Jean-Pierre Quenot, Marine Jacquier, Isabelle Fournel, Nicolas Meunier-Beillard, Clotilde Grangé, Fiona Ecarnot, Marie Labruyère, Jean-Philippe Rigaud, the RESC Study group

**Affiliations:** 1 Service de Médecine Intensive-Réanimation, CHU Dijon-Bourgogne, Bourgogne, France; 2 INSERM, CIC 1432, Module Epidémiologie Clinique, Dijon, France; 3 CHU Dijon-Bourgogne, Centre d’Investigation Clinique, Module Epidémiologie Clinique/Essais Cliniques, Dijon, France; 4 Equipe Lipness, Centre de Recherche INSERM UMR1231 et LabEx LipSTIC, Université de Bourgogne-Franche Comté, Dijon, France-INSERM; 5 Espace de Réflexion Éthique Bourgogne Franche-Comté (EREBFC), Dijon, France; 6 DRCI, USMR, CHU Dijon Bourgogne, Bourgogne, France; 7 EA3920, Department of Cardiology, University Hospital Besancon, Besancon, France; 8 Department of Intensive Care, Centre Hospitalier de Dieppe, Dieppe, France; 9 Espace de Réflexion Éthique de Normandie, University Hospital Caen, Caen, France; Cairo University, EGYPT

## Abstract

**Introduction:**

In a nationwide survey of practices, we sought to define the criteria, circumstances and consequences of non-beneficial admissions to the intensive care unit (ICU), with a view to proposing measures to avoid such situations.

**Methods:**

ICU physicians from a French research in ethics network participated in an online survey. The first part recorded age, sex, and years’ experience of the participants. In the second part, there were 8 to 12 proposals on each of 4 main domains: (1) What criteria could be used to qualify an ICU stay as non-beneficial? (2) What circumstances result in the admission of a patient whose ICU stay may later be deemed non-beneficial? (3) What are the consequences of a non-beneficial stay in the ICU? (4) What measures could be implemented to avoid admissions that later come to be considered as non-beneficial? Responses were on a 5-point Likert scale ranging from “Strongly disagree” to “Strongly agree”.

**Results:**

Among 164 physicians contacted, 154 (94%) responded. The majority cited several criteria used to qualify a stay as non-beneficial. Similarly, >80% cited several possible circumstances that could result in non-beneficial admissions, including lack of knowledge of the case and the patient’s history, and failure to anticipate acute deterioration. Possible consequences of non-beneficial stays included stress and anxiety for the patient/family, misunderstandings and conflict. Discussing the utility of possible ICU admission in the framework of the patient’s overall healthcare goals was hailed as a means to prevent non-beneficial admissions.

**Conclusion:**

The results of this survey suggest that joint discussions should take place during the patient’s healthcare trajectory, before the acute need for ICU arises, with a view to limiting or avoiding ICU stays that may later come to be deemed “non-beneficial”.

## Introduction

The COVID-19 pandemic has prompted profound reflection about the distribution of healthcare, notably that most precious of resources, the intensive care unit (ICU) bed. In particular, there has been ethical reflection on the appropriate criteria for admission to the ICU [1,2]. It behoves the ICU physician to strive to guarantee equitable utilization of the available ICU beds, and to ensure that the care dispensed is proportional and in line with the patient’s healthcare goals and wishes (when they are known), with a view to maintaining an acceptable quality of life (both mental and physical) after the ICU stay [3,4]. However, despite these well-recognized criteria that enable ICU physicians to make admission decisions to the ICU, difficulties can sometimes arise. In particular, difficulties arise in emergency situations, where the admitting physician must find the right balance between the potential loss of opportunity if admission is refused, and the risk of unreasonable medical obstinacy, if the admission is accepted but turns out to be non-beneficial for the patient. This delicate equilibrium is discussed in the guidelines of the Society of Critical Care Medicine [5,6]. In the literature, there has been much debate and discussion surrounding the use of terms such as “futile”, “inappropriate” or “excessive” [7–9], and the term “non-beneficial” has also been proposed [10]. Among the various terms used, it has been asserted that “non-beneficial” has less threatening connotations than “futile” and suggests a more professional estimation of what is in the patient’s best interests [5]. In one North-American study, 31% of patients who died in hospital were admitted to the ICU, despite the fact that they had clearly expressed a desire to receive comfort care only, and among these, 14% had received one or more life-sustaining therapies [11]. Recently, a prospective, multicentre study from France involving 4 centres showed that up to 22% of ICU stays could be deemed “non-beneficial” [12]. The authors showed that in approximately one quarter of “non-beneficial” ICU admissions, the patient had not wanted to be admitted to the ICU. Similarly, factors such as age, autonomy and previous quality of life, as well as terminal stage disease and a lack of curative therapeutic options were the factors reported by physicians to be at the origin of non-beneficial ICU stays.

In a nationwide survey of practices, we sought to define the criteria, circumstances and consequences of “non-beneficial” admissions to the ICU, with a view to proposing measures to avoid such situations. This survey was performed through the French network for ethics research in critical care (Réseau de Recherche en Éthique en Soins Critiques, RESC), to ensure representative recruitment of centres with varying specialties, size and work practices.

## Materials and methods

The survey was carried out between 22/06/2021 and 4/10/2021. All the members of the RESC network (N = 164; all ICU physicians) were invited by email to participate in the present study, and those who accepted were sent a personal code to access the survey online (via the LimeSurvey platform). No compensation of any kind was given for participation. The survey first recorded demographic characteristics of the participant (age, sex, number of years of experience as an ICU physician). Then, there were 2 questions about the ICU where the physician worked, namely: (1) What proportion of stays in your ICU are “non-beneficial” each year, in your estimation? (2) Among the “non-beneficial” stays, what proportion of them could have been avoided, in your opinion? The respondent had the possibility to choose an estimated proportion from among the following: 0 to 10%, 11 to 20%, 21 to 30% and >30% for question 1, and 0 to 25%, 26 to 50%, 51 to 75%, 76 to 100% for question 2.

Next, the participant was invited to complete a second questionnaire covering 4 main domains. Respondents were asked to rate their level of agreement on a 5-point Likert scale (Strongly disagree, Disagree, Neutral, Agree, Strongly agree) with each of the propositions in each domain. The domains were as follows: (1) What criteria could be used to qualify an ICU stay as “non-beneficial”? (8 propositions); (2) What kind of circumstances can result in the admission of a patient whose ICU stay will ultimately come to be deemed “non-beneficial”? (12 propositions); (3) What are the possible consequences of a “non-beneficial” stay in the ICU? (9 propositions); and (4) What measures could be implemented to avoid admissions that later come to be considered as “non-beneficial”? (9 propositions). In each domain, there was also an additional option entitled “Other”, where respondents could add free text comments. The English translation of the full questionnaire is provided in S1 File. Only questionnaires where the participant read through to the end were considered for analysis.

The 4 domains addressed by the questionnaire, as well as the individual questions in each domain were developed based on a focus group of 10 physicians from university and non-academic hospitals, according to the methodology previously described elsewhere [12]. Briefly, 1 clinician (JPQ) and 1 sociologist (NMB) led the focus group discussions using open-ended questions to prompt the participants to envisage all the aspects that contribute to an ICU stay being considered as inappropriate or non-beneficial. Proposals for the questionnaire items were circulated to group members and a final, consensual version of the questionnaire was achieved through a modified Delphi method. Then, the questionnaire was pilot-tested on a panel of 10 ICU physicians to assess its comprehensibility and the relevance of each item, as well as the reproducibility of responses (test/retest). Rephrasing and specification was performed until validation of the final version of the questionnaire, which was then made available online for the study participants. The panel of ICU physicians who pilot-tested the questionnaire were not respondents on the final version.

The online management of the questionnaire, data collection and analysis was performed by the Clinical Investigation Centre of the University Hospital of Dijon (certified ISO 9001 V2015). For the purposes of reporting, the response modalities “strongly agree” and “agree” were grouped, as were the options “strongly disagree” and “disagree”.

The Institutional Review Board (Comité de Protection des Personnes Est I, Dijon) declared that this study falls outside the scope of legislation governing clinical research in France, as it involves healthcare professionals. Each participant nonetheless provided consent to participate after viewing the study information at the beginning of the survey.

## Results

Among the 164 members of the RESC network who were initially invited, 154 accepted to participate and completed the questionnaires. Three-quarters of them had more than 2 years’ experience as ICU physicians. Only 7% of physicians responded that more than 21% of stays in their ICU per year could be considered as “non-beneficial”, and one third estimated that half of these “non-beneficial” stays could be avoided. The characteristics of the study population are presented in S1 Table in S2 File. There was no significant difference in responses according to sex or number of years’ experience working in the ICU.

### Question 1: What criteria could be used to qualify an ICU stay as “non-beneficial”? (Fig 1)

**Fig 1:**
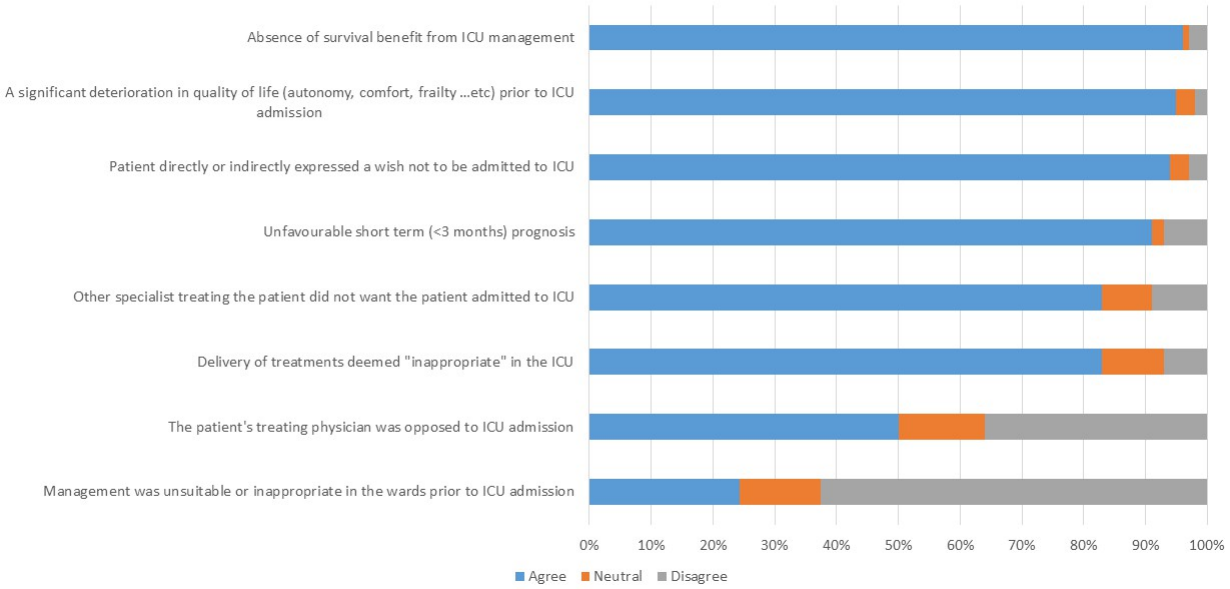
Level of reported agreement with the criteria used to qualify an ICU stay as “non-beneficial” (Domain 1 of the questionnaire).

The majority (>80%) of respondents cited several criteria that could be used to deem an ICU stay as “non-beneficial”. These included: absence of survival benefit from ICU management, a significant deterioration in quality of life, the wishes expressed by the patient, unfavourable short-term prognosis (<3 months), the wishes expressed by the patient’s other treating physicians, and the delivery of treatments deemed inappropriate in the ICU. Conversely, unsuitable or suboptimal management prior to the ICU admission was not widely cited as a criterion for judging the ICU stay to be “non-beneficial”.

The free text comments for all four questions are analysed and provided in S3 File.

### Question 2: What kind of circumstances can result in the admission of a patient whose ICU stay will ultimately come to be deemed “non-beneficial”? (Fig 2)

**Fig 2:**
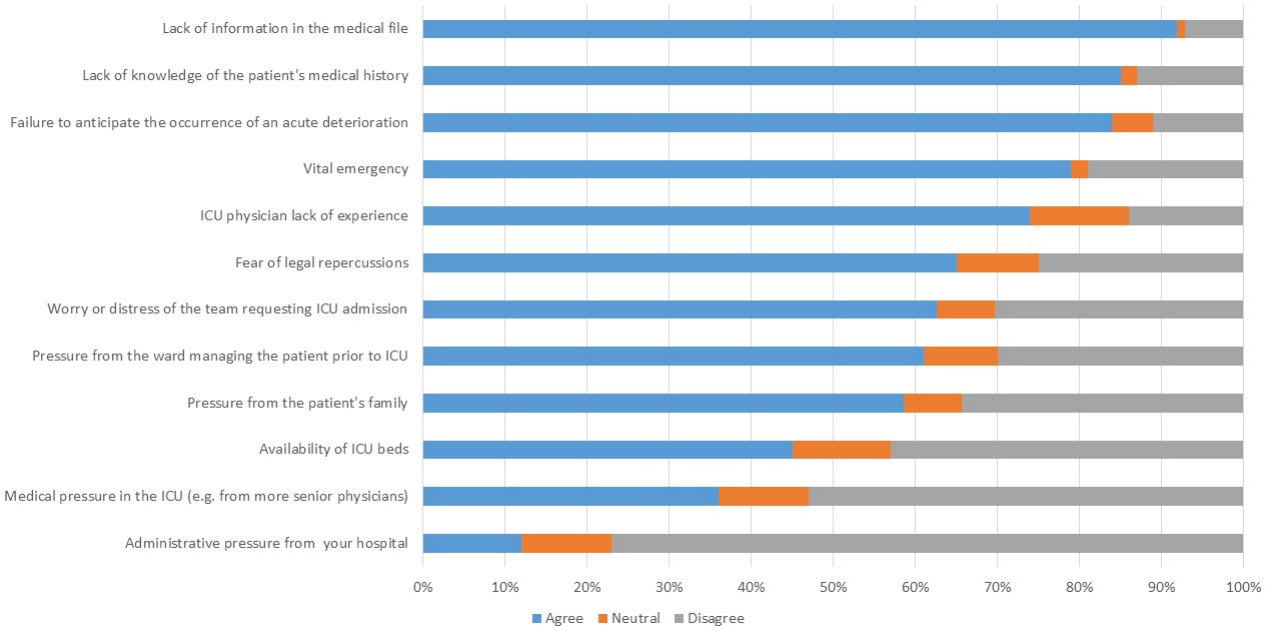
Level of reported agreement with the different circumstances that could result in the admission of a patient whose ICU stay will ultimately come to be deemed “non-beneficial” (Domain 2 of the questionnaire).

Again, the majority (80%) cited several possible circumstances that could result in an admission that could subsequently be deemed “non-beneficial”, such as: a lack of information, lack of knowledge of the patient’s medical history, failure to anticipate an acute deterioration. The availability of ICU beds and pressure from other physicians or the administration were not widely cited as concerns leading to a “non-beneficial” admission.

### Question 3: What are the possible consequences of a “non-beneficial” stay in the ICU? (Fig 3)

**Fig 3:**
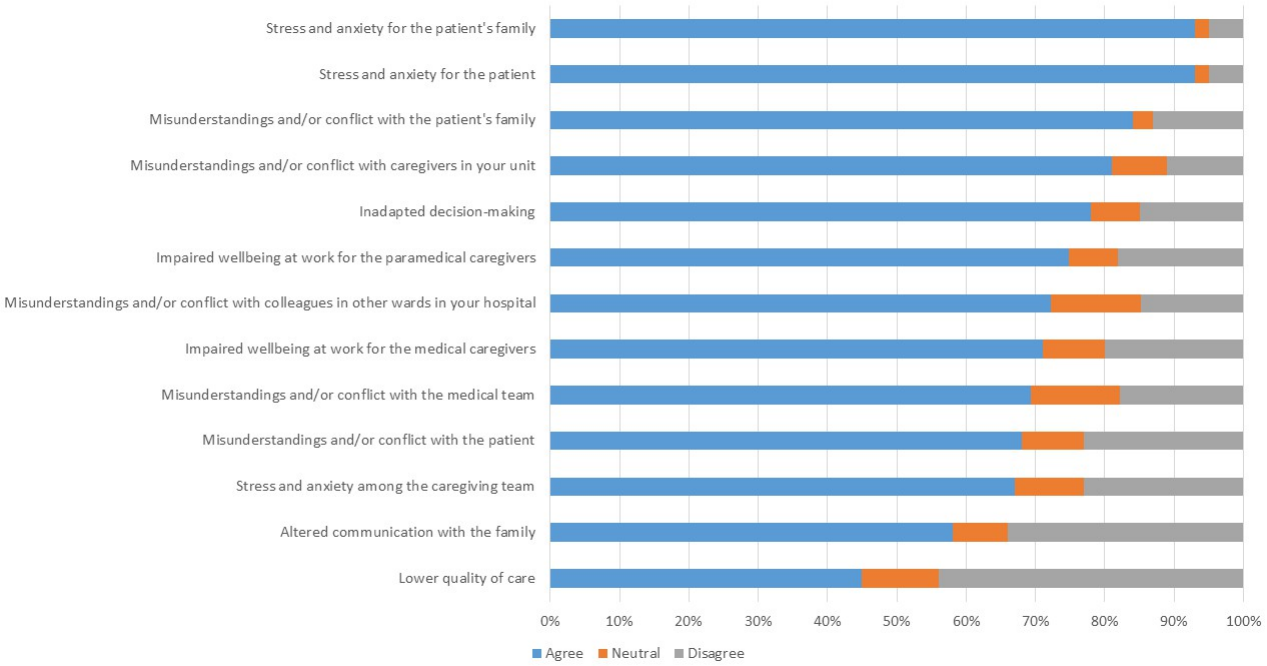
Level of reported agreement with the different possible consequences of a “non-beneficial” stay in the ICU (Domain 3 of the questionnaire).

Again, more than 80% of respondents cited several possible consequences of a “non-beneficial” stay in the ICU, including: stress and anxiety for the patient and family, misunderstandings and/or conflict with the family or ICU caregivers. It should be noted that a lower quality of care was cited in 45% of the responses as a possible consequence of a “non-beneficial” admission.

### Question 4: What measures could be implemented to avoid admissions that later come to be considered as “non-beneficial”? (Fig 4)

**Fig 4:**
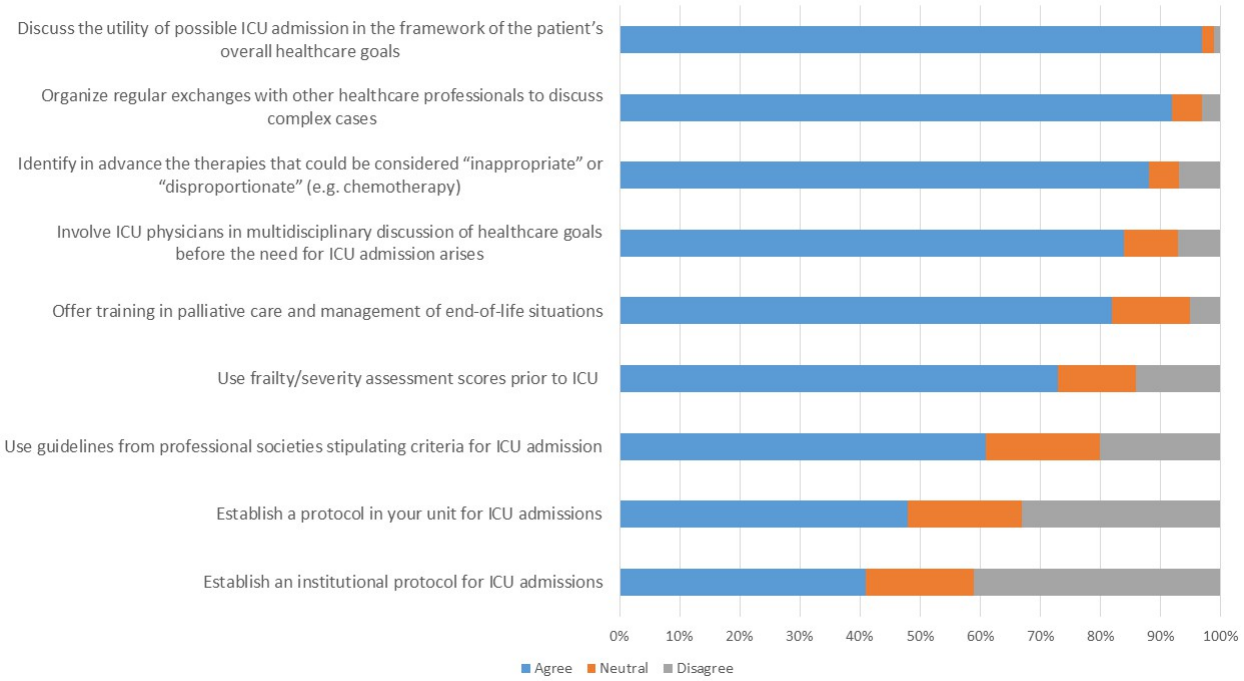
Level of reported agreement with the proposed measures to avoid admissions that later come to be considered as “non-beneficial” (Domain 4 of the questionnaire).

Finally, more than 80% of respondents proposed several measures that could be implemented to avoid “non-beneficial” ICU admissions, including the following: Discussing the utility of possible ICU admission in the framework of the patient’s overall healthcare goals; establishing an institutional protocol for decision-making about ICU admissions; identifying in advance the therapies that could be considered “inappropriate” or “disproportionate” prior to ICU admission; involving ICU physicians in the multidisciplinary development of healthcare goals for a patient before the need for ICU admission arises; organizing regular exchanges with other healthcare professionals to discuss complex cases. The use of frailty and severity scores were cited by more than 70% of respondents as measures likely to avoid “non-beneficial” ICU stays.

## Discussion

This study highlights the range of considerations that come into play when deciding on admission of a patient to the ICU. Our respondents were predominantly in agreement with the proposed circumstances that may identify a potentially non-beneficial ICU stay, as well as the consequences of, and measures that might be taken to limit or avoid non-beneficial stays. To the best of our knowledge, this is the first survey of practices performed in this area. The main results underline the fact that admission to the ICU should take account of expected survival and quality of life beyond the ICU, in light of the patient’s current and prior healthcare pathway. Clinical emergencies, a lack of knowledge about the patient’s medical history, and failure to anticipate ICU admission in the patient’s healthcare goals are all circumstances that may culminate in a non-beneficial ICU admission. Stress, anxiety, misunderstandings or even conflict between staff and families may result from such inopportune admissions. Finally, one of the measures suggested in our study as a potential means to limit or avoid unpropitious admissions was multidisciplinary discussion before the need for ICU care arises. These discussions should involve the patient and family, and an ICU physician, if needed, depending on the patient’s healthcare goals.

In practice, the question arises as to how we can take action in light of these results, which closely reflect the situations that ICU physicians experience on a daily basis when having to choose whether or not to admit a patient to the ICU. The intensivist has a moral duty with regard to the delivery of healthcare–indeed, care that may often be burdensome for the patient, the family and the caregivers. Furthermore, ICU care is costly, in terms of human, material and financial resources, in a healthcare system where funds are increasingly constrained [13]. The ICU physician’s moral duty must be considered in context: often, the intensivist is called on in an emergency, has little or no medical knowledge of the specific case on which to judge whether ICU admission is appropriate, or on which to base a decision to introduce life-support therapy. In all likelihood, this weighty responsibility should be shared with other physicians earlier in the patient’s healthcare trajectory, before the need for ICU admission arises, and involving all the treating physicians. In spite of this, non-ICU physicians argue that there are numerous obstacles to anticipating need for ICU care, such as the uncertain prognosis in many patients [14–16], insufficient hindsight regarding the patient’s clinical condition, prior history, quality of life, and healthcare goals, as well as difficulties informing patients and their families about the potential occurrence of an acute decompensation, and the lack of knowledge about the specific care processes available in the ICU [17]. To overcome these difficulties, several approaches have been proposed, such as advance care planning (ACP) [18,19], ethics consultations and palliative care consultations [20–22]. These approaches generally lead to a reduction in disproportionate care delivered in the ICU. One should also remember that many patients find it hard to express their wishes, even when the legislation offers the possibility to lay down advance directives, as is the case in France [23] and internationally [24,25]. For many patients, it is challenging to seize this opportunity to formally prepare advance directives, even after they have been hospitalized in the ICU. This is likely because most people have difficulty imagining their own end-of-life, and envisaging the circumstances and decisions that go with it [26].

One interesting finding is that some respondents admit patients to the ICU for fear of possible legal repercussions if they do not do so. This could be considered akin to “defensive medicine”. Defensive medicine is the practice of deviating from standard medical practice in the aim of avoiding potential criticism, legal repercussions, or loss of reputation. Some of the potential consequences of this practice include providing unnecessary examinations or excessively aggressive care, incurring avoidable human and economic costs [27]. In France, the legislation imposes an obligation to commit the appropriate means (i.e. resources), but there is no legal obligation in terms of results. In this context, many physicians may prefer to commit the appropriate means, and admit a patient to ICU, thereby avoiding potential loss-of-opportunity to the patient, and possible legal repercussions for themselves, even if there is the possibility that this will later be deemed to have been “non-beneficial”. A recent narrative review of medical errors, medical negligence and defensive medicine found that inadequate availability of patient information was a leading cause of medical error, and concluded that the essential attitude to avoid medical liability is a good and ethical medical practice with the proper use of technology, based on knowledge of scientific evidence and ethical principles of medicine for the benefit of patients [28]. We would add that that this attitude should also be based on adequate knowledge of the patient’s medical history, personal values and goals of care, to ensure the best decisions are made for each patient.

In view of the issues brought to light by this survey, which collectively may culminate in non-beneficial ICU admissions, we propose that there should be multidisciplinary interaction and discussion prior to the need for ICU admission, about the level of therapeutic engagement that is appropriate for each patient. These discussions should involve the patient and family, and the treating physicians, and could also call on an ICU physician as a key consultant to inform about the possibilities for ICU admission, as previously suggested by Hilton et al [29]. Indeed, the ICU physician knows best what types of life-support or other therapies can be provided in the ICU. The intensivist is also best placed to evaluate the patient’s prognosis, according to the presence and/or severity of organ failure. Finally, the ICU physician is also best qualified to explain to the patient and family what ICU care can or cannot achieve, and what the possible consequences of a stay in the ICU might be. Several previous publications have highlighted the importance of communication and discussions surrounding goals of care in critically ill patients [30–33]. The majority of the sample of French ICU practitioners surveyed in this study were in agreement with this, yet it is telling that they continue to report this as an unmet need, highlighting the fact that there remains room for improvement in terms of goals-of-care discussions, both before and during ICU admission.

This study has several strengths, including the representativeness of the centres in terms of the modes of practice of ICU physicians. The proportion of respondents was very high compared to what is usually reported in the literature. Finally, the methodology, especially the development of the questionnaire based on focus groups and a pilot panel, are a guarantee of the robustness of the results.

Conversely, this study also has some limitations. Firstly, the absolute number of participating ICU physicians is relatively low, but is similar to the numbers reported in other surveys on similar topics [34,35]. Second, physicians who volunteered to respond may be particularly motivated, since they were all members of a research network. In addition, the target population were members of a research network in ethics, which may limit the generalizability of the findings to physicians from other backgrounds or working in other settings. Third, there may be some potential for social desirability bias, whereby the respondents provided the answer they thought would be most acceptable, rather than what they actually do or think in practice. As with all studies of self-reported practices, we cannot be 100% certain that the reported practices actually correspond to what is done in reality at the patient’s bedside. Similarly, self-reported estimations, such as the proportion of non-beneficial admissions per year, should be interpreted with caution. Finally, responses are approximated through a Likert scale, and therefore, the granularity and nuances of the answers warrant further investigation in real-life practice at the bedside. In terms of perspectives, other stakeholder groups, such as the patients, their families, nursing staff or physicians from other specialities also warrant investigation in future studies.

## Conclusion

The results of this survey of practices administered online suggest that joint discussions should take place during the patient’s healthcare trajectory, before the acute need for ICU arises, with a view to limiting or avoiding ICU stays that may later come to be deemed “non-beneficial”. This could be achieved via greater implication of ICU physicians in discussions pertaining to patients hospitalized in wards other than the ICU, in collaboration with non-ICU physicians, to identify and anticipate which patients would benefit most from ICU care, in the context of their healthcare goals. Interventional studies are warranted in the future to test the efficacy of the measures proposed in this article.

## Supporting information

S1 File English translation of the questionnaire. (DOC)

S2 File Supplementary Table detailing the characteristics of the study population. (DOCX)

S3 File Free text comments for all four questions from the questionnaire. (DOCX)

## References

[1] Lesieur O, Quenot JP, Cohen-Solal Z, David R, De Saint Blanquat L, Elbaz M, et al. Admission criteria and management of critical care patients in a pandemic context: position of the Ethics Commission of the French Intensive Care Society, update of April 2021. Ann Intensive Care. 2021;11:66. Epub 20210426. doi: 10.1186/s13613-021-00855-z 33904016 PMC8076390

[2] Robert R, Kentish-Barnes N, Boyer A, Laurent A, Azoulay E, Reignier J. Ethical dilemmas due to the Covid-19 pandemic. Ann Intensive Care. 2020;10:84. Epub 20200617. doi: 10.1186/s13613-020-00702-7 32556826 PMC7298921

[3] Amin P, Fox-Robichaud A, Divatia JV, Pelosi P, Altintas D, Eryuksel E, et al. The Intensive care unit specialist: Report from the Task Force of World Federation of Societies of Intensive and Critical Care Medicine. J Crit Care. 2016;35:223–8. Epub 20160621. doi: 10.1016/j.jcrc.2016.06.001 27444985

[4] Quenot JP, Ecarnot F, Meunier-Beillard N, Dargent A, Large A, Andreu P, et al. What are the ethical aspects surrounding the collegial decisional process in limiting and withdrawing treatment in intensive care? Ann Transl Med. 2017;5:S43. doi: 10.21037/atm.2017.04.15 29302599 PMC5750242

[5] Nates JL, Nunnally M, Kleinpell R, Blosser S, Goldner J, Birriel B, et al. ICU Admission, Discharge, and Triage Guidelines: A Framework to Enhance Clinical Operations, Development of Institutional Policies, and Further Research. Crit Care Med. 2016;44:1553–602. doi: 10.1097/CCM.0000000000001856 27428118

[6] Sprung CL, Danis M, Iapichino G, Artigas A, Kesecioglu J, Moreno R, et al. Triage of intensive care patients: identifying agreement and controversy. Intensive Care Med. 2013;39:1916–24. Epub 20130808. doi: 10.1007/s00134-013-3033-6 23925544 PMC5549951

[7] Kubler A, Siewiera J, Durek G, Kusza K, Piechota M, Szkulmowski Z. Guidelines regarding the ineffective maintenance of organ functions (futile therapy) in ICU patients incapable of giving informed statements of will. Anaesthesiol Intensive Ther. 2014;46:215–20. 25293473 10.5603/AIT.a2014.0038

[8] Piers RD, Azoulay E, Ricou B, Dekeyser Ganz F, Decruyenaere J, Max A, et al. Perceptions of appropriateness of care among European and Israeli intensive care unit nurses and physicians. JAMA. 2011;306:2694–703. doi: 10.1001/jama.2011.1888 22203538

[9] Benoit DD, Jensen HI, Malmgren J, Metaxa V, Reyners AK, Darmon M, et al. Outcome in patients perceived as receiving excessive care across different ethical climates: a prospective study in 68 intensive care units in Europe and the USA. Intensive Care Med. 2018;44:1039–49. doi: 10.1007/s00134-018-5231-8 29808345 PMC6061457

[10] Close E, Parker M, Willmott L, White B, Crowden A. Australian Policies on "Futile" or "Non-beneficial" Treatment at the End of Life: A Qualitative Content Analysis. J Law Med. 2019;27:415–39. 32129045

[11] Lee RY, Brumback LC, Sathitratanacheewin S, Lober WB, Modes ME, Lynch YT, et al. Association of Physician Orders for Life-Sustaining Treatment With ICU Admission Among Patients Hospitalized Near the End of Life. JAMA. 2020;323:950–60. doi: 10.1001/jama.2019.22523 32062674 PMC7042829

[12] Quenot JP, Large A, Meunier-Beillard N, Pugliesi PS, Rollet P, Toitot A, et al. What are the characteristics that lead physicians to perceive an ICU stay as non-beneficial for the patient? PLoS One. 2019;14:e0222039. doi: 10.1371/journal.pone.0222039 31490986 PMC6730882

[13] Wallace SK, Rathi NK, Waller DK, Ensor JE Jr., Haque SA, Price KJ, et al. Two Decades of ICU Utilization and Hospital Outcomes in a Comprehensive Cancer Center. Crit Care Med. 2016;44:926–33. doi: 10.1097/CCM.0000000000001568 26765498

[14] Gibbins J, McCoubrie R, Alexander N, Kinzel C, Forbes K. Diagnosing dying in the acute hospital setting—are we too late? Clin Med (Lond). 2009;9:116–9. Epub 2009/05/14. doi: 10.7861/clinmedicine.9-2-116 19435113 PMC4952659

[15] Glare P, Virik K, Jones M, Hudson M, Eychmuller S, Simes J, et al. A systematic review of physicians’ survival predictions in terminally ill cancer patients. BMJ. 2003;327:195–8. Epub 2003/07/26. [doi] 327/7408/195 [pii]. doi: 10.1136/bmj.327.7408.195 12881260 PMC166124

[16] Finucane TE. How gravely ill becomes dying: a key to end-of-life care. JAMA. 1999;282:1670–2. Epub 1999/11/30. jed90078 [pii]. doi: 10.1001/jama.282.17.1670 10553796

[17] Puchalski CM, Zhong Z, Jacobs MM, Fox E, Lynn J, Harrold J, et al. Patients who want their family and physician to make resuscitation decisions for them: observations from SUPPORT and HELP. Study to Understand Prognoses and Preferences for Outcomes and Risks of Treatment. Hospitalized Elderly Longitudinal Project. J Am Geriatr Soc. 2000;48:S84–90. doi: 10.1111/j.1532-5415.2000.tb03146.x 10809461

[18] Khandelwal N, Kross EK, Engelberg RA, Coe NB, Long AC, Curtis JR. Estimating the effect of palliative care interventions and advance care planning on ICU utilization: a systematic review. Crit Care Med. 2015;43:1102–11. Epub 2015/01/13. [doi]. doi: 10.1097/CCM.0000000000000852 25574794 PMC4499326

[19] Quenot JP, Ecarnot F, Meunier-Beillard N, Dargent A, Large A, Andreu P, et al. What are the ethical questions raised by the integration of intensive care into advance care planning? Ann Transl Med. 2017;5:S46. doi: 10.21037/atm.2017.08.08 29302602 PMC5750251

[20] Schneiderman LJ, Gilmer T, Teetzel HD, Dugan DO, Blustein J, Cranford R, et al. Effect of ethics consultations on nonbeneficial life-sustaining treatments in the intensive care setting: a randomized controlled trial. JAMA. 2003;290:1166–72. Epub 2003/09/04. [doi] 290/9/1166 [pii]. doi: 10.1001/jama.290.9.1166 12952998

[21] Molloy DW, Guyatt GH, Russo R, Goeree R, O’Brien BJ, Bedard M, et al. Systematic implementation of an advance directive program in nursing homes: a randomized controlled trial. JAMA. 2000;283:1437–44. Epub 2000/03/25. joc90890 [pii]. doi: 10.1001/jama.283.11.1437 10732933

[22] Quill TE, Holloway R. Time-limited trials near the end of life. JAMA. 2011;306:1483–4. Epub 2011/10/06. 306/13/1483 [pii] [doi]. doi: 10.1001/jama.2011.1413 21972312

[23] Law 2016–87 dated 2 February 2016 introducing new rights for patients and persons at the end-of-life. Journal Officiel de la Republique Francaise; 3 February 2016. https://www.legifrance.gouv.fr/affichTexte.do;jsessionid=46038F123BBBFC8DA2E8DE0EEE161860.tpdila19v_1?cidTexte=JORFTEXT000031970253&categorieLien=id (access date: 30 March 2018); 2016.

[24] Myburgh J, Abillama F, Chiumello D, Dobb G, Jacobe S, Kleinpell R, et al. End-of-life care in the intensive care unit: Report from the Task Force of World Federation of Societies of Intensive and Critical Care Medicine. J Crit Care. 2016;34:125–30. doi: 10.1016/j.jcrc.2016.04.017 27288625

[25] Sprung CL, Truog RD, Curtis JR, Joynt GM, Baras M, Michalsen A, et al. Seeking worldwide professional consensus on the principles of end-of-life care for the critically ill. The Consensus for Worldwide End-of-Life Practice for Patients in Intensive Care Units (WELPICUS) study. Am J Respir Crit Care Med. 2014;190:855–66. Epub 2014/08/28. [doi]. doi: 10.1164/rccm.201403-0593CC 25162767

[26] Andreu P, Dargent A, Large A, Meunier-Beillard N, Vinault S, Leiva-Rojas U, et al. Impact of a stay in the intensive care unit on the preparation of Advance Directives: Descriptive, exploratory, qualitative study. Anaesth Crit Care Pain Med. 2018;37:113–9. doi: 10.1016/j.accpm.2017.05.007 28826983

[27] Kattel P. Defensive Medicine: Is It Legitimate or Immoral? J Nepal Health Res Counc. 2019;16:483–5. Epub 20190128. 30739921

[28] Miziara ID, Miziara C. Medical errors, medical negligence and defensive medicine: A narrative review. Clinics (Sao Paulo). 2022;77:100053. Epub 20220528. doi: 10.1016/j.clinsp.2022.100053 35640458 PMC9160317

[29] Hilton AK, Jones D, Bellomo R. Clinical review: the role of the intensivist and the rapid response team in nosocomial end-of-life care. Crit Care. 2013;17:224. Epub 2013/05/16. cc11856 [pii] [doi]. doi: 10.1186/cc11856 23672813 PMC3672544

[30] Curtis JR, Downey L, Back AL, Nielsen EL, Paul S, Lahdya AZ, et al. Effect of a Patient and Clinician Communication-Priming Intervention on Patient-Reported Goals-of-Care Discussions Between Patients With Serious Illness and Clinicians: A Randomized Clinical Trial. JAMA Intern Med. 2018;178:930–40. doi: 10.1001/jamainternmed.2018.2317 29802770 PMC6145723

[31] Zhang B, Wright AA, Huskamp HA, Nilsson ME, Maciejewski ML, Earle CC, et al. Health care costs in the last week of life: associations with end-of-life conversations. Arch Intern Med. 2009;169:480–8. doi: 10.1001/archinternmed.2008.587 19273778 PMC2862687

[32] Wright AA, Zhang B, Ray A, Mack JW, Trice E, Balboni T, et al. Associations between end-of-life discussions, patient mental health, medical care near death, and caregiver bereavement adjustment. JAMA. 2008;300:1665–73. doi: 10.1001/jama.300.14.1665 18840840 PMC2853806

[33] Ryan RE, Connolly M, Bradford NK, Henderson S, Herbert A, Schonfeld L, et al. Interventions for interpersonal communication about end of life care between health practitioners and affected people. Cochrane Database Syst Rev. 2022;7:CD013116. Epub 20220708. doi: 10.1002/14651858.CD013116.pub2 35802350 PMC9266997

[34] Rigaud JP, Moutel G, Quesnel C, Eraldi JP, Bougerol F, Pavon A, et al. How patient families are provided with information during intensive care: A survey of practices. Anaesth Crit Care Pain Med. 2016;35:185–9. Epub 2016/03/24. S2352-5568(16)30015-7 [pii] [doi]. doi: 10.1016/j.accpm.2016.03.002 27004918

[35] Rigaud JP, Giabicani M, Meunier-Beillard N, Ecarnot F, Beuzelin M, Marchalot A, et al. Non-readmission decisions in the intensive care unit under French rules: A nationwide survey of practices. PLoS One. 2018;13:e0205689. doi: 10.1371/journal.pone.0205689 30335804 PMC6193659

