# Supplementary material for: Non-beneficial admission to the intensive care unit: A nationwide survey of practices
Source: PLoS One. 2023 Feb 2;18(2):e0279939. doi: 10.1371/journal.pone.0279939 (PMC9894425; doi:10.1371/journal.pone.0279939)
Supplement: S1 File — (DOC) [file pone.0279939.s001.doc]

**Survey of Practices: ICUs stays that are deemed non-beneficial**

Dear Colleagues,

In our daily practice, admission to the ICU usually poses no particular ethical dilemmas. This is the case, for example, when the ICU admission has curative intent, and intensive care is delivered with maximal therapeutic engagement, in the aim of guaranteeing survival and quality of life in line with the patient’s values and preferences. However, in certain circumstances, there may be some ambiguity about the necessity or justification for ICU admission. These ambiguities arise mainly in situations of unplanned admission, where the possibility of admission has not been anticipated, and consequently, has not been discussed. These admissions may later come to be considered as non-beneficial for the patient.

In this context, we are performing a survey of practices to find out what you think about non-beneficial stays in your ICU.

**The characteristics of each participating ICU are recorded via a specific questionnaire sent previously.**

**What age are you ?**

**<35 years 36-50 years >51 years**

☐ ☐ ☐

**How long have you been working in the ICU?**

< 5 years 5 to 10 years more than 10 years

☐ ☐ ☐

**Among all the patients admitted to your ICU annually, please give an estimate of the proportion of stays that are non-beneficial in your opinion?**

From 0% to 10% from 11 to 20% from 21 to 30% > 30%

☐ ☐ ☐ ☐

**Among these non-beneficial stays, what proportion of them could have been avoided in your opinion?**

0% to 25 % 26 to 50% 51% to 75% 76% to 100%

☐ ☐ ☐ ☐

1. **For the following CRITERIA that could be used to classify ICU stays as non-beneficial, please indicate your level of agreement with each statement:**

☐ Strongly disagree ☐ Disagree ☐ Neutral ☐ Agree ☐ Strongly agree

- The patient directly or indirectly expressed a wish not to be admitted to ICU (to a family member, relative, surrogate, in advance directives…etc)
- The patient’s treating physician did not want the patient to be admitted to ICU
- The specialists taking care of the patient did not want the patient to be admitted to ICU
- There was a significant decline in quality of life (autonomy, comfort, frailty, etc) prior to the ICU stay
- The patient had a short life expectancy (<3 months)
- ICU was not likely to yield any benefit for the patient in terms of survival and/or acceptable quality of life
- The care delivered in ICU is inappropriate for this patient.
- The care delivered prior to ICU admission was inappropriate or insufficient.
- Other: please specify____________________________________________

1. **Please indicate your level of agreement with each of the following CIRCUMSTANCES that could lead to an ICU admission that might later come to be judged as non-beneficial?**

☐ Strongly disagree ☐ Disagree ☐ Neutral ☐ Agree ☐ Strongly agree

- Vital emergency
- Lack of knowledge of the medical context
- Insufficient information in the medical file
- Failure to anticipate a potential acute deterioration
- Worry and distress among the staff of the ward requesting ICU admission
- Pressure from colleagues before ICU admission
- Pressure from colleagues in the ICU (from your superior, for example)
- Pressure from the patient’s family
- Inexperience of the ICU physician
- Administrative pressure from your hospital
- Availability of beds in the ICU
- Fear of legal repercussions
- Other: please specify____________________________________________

1. **Please indicate your level of agreement with each of the following possible CONSEQUENCES of a non-beneficial ICU stay?**

☐ Strongly disagree ☐ Disagree ☐ Neutral ☐ Agree ☐ Strongly agree

- Stress and anxiety for the patient
- Stress and anxiety for the patient’s family
- Lower quality of care
- Inadapted medical decisions
- Stress and anxiety for the caregiving staff
- Altered work quality for the paramedical staff
- Altered work quality for the medical staff
- Impaired communication with the family
- Situations of conflict or misunderstanding with….
  - The patient
  - The family
  - Colleagues in the ICU
  - Colleagues in other wards of the hospital
  - Caregiving staff in the ICU
  - Members of the medical team
- Other: please specify____________________________________________

1. **Please indicate your level of agreement with the following MEASURES that could be implemented to avoid admissions that come to be deemed non-beneficial after the fact:**

☐ Strongly disagree ☐ Disagree ☐ Neutral ☐ Agree ☐ Strongly agree

- Discuss the value of ICU admission in the framework of the patient’s overall healthcare goals
- Invite an ICU physician to participate in multidisciplinary discussions of the patient’s healthcare goals, prior to the need for ICU admission
- Identify and specify treatment(s) that would be considered inappropriate or disproportionate prior to need for ICU (e.g. chemotherapy)
- Use scores of severity and/or frailty prior to need for ICU admission
- Implement a standardized procedure for ICU admissions in your unit
- Establish an institution-level procedure for ICU admission
- Organize regular exchanges with other health professionals about complex healthcare situations (e.g. debriefing, analysis of errors, multidisciplinary discussions, morbi-mortality review, ethics meetings etc)
- Offer training in palliative care and management of end-of-life situations
- Designate a reference person for ICU admission criteria, based on recommendations from professional societies
- Other: please specify____________________________________________
