# Supplementary material for: Non-beneficial admission to the intensive care unit: A nationwide survey of practices
Source: PLoS One. 2023 Feb 2;18(2):e0279939. doi: 10.1371/journal.pone.0279939 (PMC9894425; doi:10.1371/journal.pone.0279939)
Supplement: S2 File — (DOCX) [file pone.0279939.s002.docx]

**Supplementary Table 1: Characteristics of the study respondents and their institutions**

| **Characteristic** | **N (%)** |
| --- | --- |
| Sex |  |
| Men | 103 (67%) |
| Women | 48 (31%) |
| Missing data | 3 (2%) |
| Age |  |
| 35 to 49 years | 67 (44%) |
| 50 years or over | 24 (15%) |
| Missing data | 63 (41%) |
| Seniority in the ICU |  |
| <5 years experience | 58 (38%) |
| 5 to 10 years experience | 38 (25%) |
| >10 years experience | 55 (36%) |
| Missing data | 3 (1%) |
| Type of hospital |  |
| Academic teaching hospital | 66 (43%) |
| Non-academic public hospital | 66 (43%) |
| Private-public partnership | 7 (4%) |
| Private clinic | 8 (5%) |
| Other | 4 (3%) |
| Missing data | 3 (2%) |
| Type of ICU |  |
| Medical | 51 (34%) |
| Surgical | 9 (6%) |
| Polyvalent | 87 (57%) |
| Paediatric | 1 (0.5%) |
| Other | 3 (2%) |
| Missing data | 1 (0.5%) |
| Annual estimated proportion of non-beneficial stays |  |
| 0% to 10% | 92 (61%) |
| 11% to 20% | 46 (30%) |
| 21% to 30% | 6 (4%) |
| >30% | 4 (2.5%) |
| Missing data | 4 (2.5%) |
| Annual estimated proportion of non-beneficial stays that could have been avoided |  |
| 0% to 25% | 50 (32.5%) |
| 26% to 50% | 50 (32.5%) |
| 51% to 75% | 36 (23%) |
| 76% to 100% | 10 (7%) |
| Missing data | 8 (5%) |
