# Supplementary material for: Non-beneficial admission to the intensive care unit: A nationwide survey of practices
Source: PLoS One. 2023 Feb 2;18(2):e0279939. doi: 10.1371/journal.pone.0279939 (PMC9894425; doi:10.1371/journal.pone.0279939)
Supplement: S3 File — (DOCX) [file pone.0279939.s003.docx]

**Summary and Translation of the Free Text Responses in Each Domain**

**Domain 1 : CRITERIA that could be used to classify ICU stays as non-beneficial**

Among 8 free-text responses in this domain, 6 (80%) could be re-classified as circumstances leading to non-beneficial admissions (without adding any new information), and 2 (20%) correspond to items that already existed among the proposed answers.

1. Initial management by a resident, without consulting the senior physician
2. Initiation of standard techniques in a ward that is no longer able to manage them (e.g. pleural drain)
3. Futility
4. When the disease or its treatment becomes more important than the patient him/herself, that often leads to ICU stays that are hard to justify
5. Bedridden patient or incurable disease (but not presented as such at admission)
6. Diagnosis of cognitive decline properly established and documented.
7. Economic criteria: no « marker » procedures
8. Personal conviction among young patients ( for example: Jehovah’s witnesses) or related to a lack of knowledge about what ICU involves

**Domain 2: CIRCUMSTANCES that could lead to an ICU admission that might later come to be judged as non-beneficial**

Among 33 free text comments provided in this domain, 12 responses (36%) actually correspond to reformulations of the items proposed among the responses; 11 responses (33%) could be classed as admissions that are justified, 2 responses (6%) correspond to the preventive measures domain, and 8 (24%) provide insights regarding both the circumstances of admission and preventive measures.

The free text responses raise two new concepts that could be integrated into future research in this area, namely:

- Lies (or untruths by omission), whereby certain pieces of information are voluntarily withheld in order to increase the chances of the patient being admitted to ICU.
- Problems related to the patient’s environment, including human resources (medical and non-medical), bed capacity in the ICU and/or hospital, inexperience of the healthcare providers caring for the patient before ICU etc.

1. Desire/custom of never saying no among the oldest intensivists
2. Erroneous initial evaluation, with an ICU stay that was expected to be short, but ultimately turned out to be long
3. Transmission of inaccurate information by the team caring for the patient (admissions by telephone)
4. If the person is a VIP or a former doctor from the hospital
5. To compensate for insufficient availability of technical resources (recovery room not operational 24/7), refusal to admit a borderline patient to surgical ICU because they have to organize their on-call duties and can’t take care of the patient.
6. To buy time for the patient’s family to get to the hospital to see the patient, give the family time to come to terms with unexpected death
7. Desire of the team referring the patient to avoid having to deal with a situation of withholding/withdrawing therapy, leading them to hide the truth
8. Intensive management beforehand. 1. Prehospital management (e.g. intubation of a patient known to want limited therapeutic intensity ; intubation of a coma patient before doing a brain CT scan that shows irreversible damage etc); 2. Management in the operating theatre without consulting other specialists (e.g. orthopaedic surgery of a bedridden or demented patient under catecholamines in theatre; visceral surgery for advanced cancer under catecholamines; surgery in a patient with documented heart failure or respiratory insufficiency with known limitation of therapy, then unable to wean off respiratory support after surgery)
9. Inclusion in a therapeutic trial of new molecules (e.g. immunotherapy). Young age of the patient. Iatrogenic nature of the cause of the life-threatening condition.
10. Patient’s request. Erroneous information given re patient’s autonomy.
11. Basically a lack of knowledge of the medical file.
12. Implementation of a systematic pathway (e.g. after surgery)
13. Lack of interest (or lack of knowledge?) among specialists in other disciplines about overall management, you sometimes have the impression that once the patient has two organs affected, then it’s straight to the ICU because no other medical specialty can provide overall (“global”) management for the patient. “he can’t go to geriatrics because he still has an abdominal drain” but in surgery, there’s nobody available to re-assess the cardiology drugs and manage autonomy at discharge….(2) logistics problems: can’t have two units of blood in our unit, can’t go to rehabilitation because the antibiotics are too dear and they won’t have them there, you have to finish the treatment period in ICU; the patient has a tracheotomy and they don’t know how to aspirate in the other units….. (3) prolongation of a stay or unsuitable decisions sometimes linked to under-staffing, with a need for a rapid decision that often turns into “when in doubt, take the patient” (it would take too long to get the necessary information and see the family), (4) easy way out taken by some physicians: it’s easier to take the patient and then think about it and/or see the family afterwards…. A family conference to explain why you’re refusing ICU admission is longer and harder to do than just admitting the patient.
14. Sometimes the ICU team is not aware of updated recommendations (e.g. excessive admissions of patients with uncomplicated non-high-risk pulmonary embolism). Failure to “respect” scores that could orient the patient elsewhere.
15. Patient’s environment (close family members are doctors/nurses, the patient is a VIP)
16. Medical repatriation
17. Emergency department saturated ; patient overflow directed to ICU to relieve the ED even though the patient might not legitimately require ICU
18. Lack of communication with intensivists prior to the need for ICU, for patients who are hospitalized and at high risk of acute clinical decompensation
19. Lack of consensus about the utility of ICU admission
20. Lack of knowledge of the disease (cf COVID)
21. Patients with tracheostomy-ventilation at home for whom no other purely medical healthcare establishments exist
22. Bed capacity saturated in the hospital
23. Lack of knowledge among non-ICU physicians about the objectives of intensive care (help the patient to survive, with acceptable quality of life afterwards), their lack of knowledge of the complications of ICU and the factors related to poor prognosis, notably functional reserve, autonomy, frailty
24. Life-threatening emergency in hospital leading to non-beneficial stay most often results from a lack of surveillance, failure to take account of clinical deterioration, and above all, lack of medical anticipation of what to do if there’s a deterioration….. the billing system and the bed occupancy rates may tempt some physicians to admit when beds are empty…..
25. Admission at night or on the weekend when the patient’s file is not fully available (can’t contact the general practitioner or the specialist who regularly follows the patient). Lies by certain colleagues from other hospitals who exaggerate the clinical picture to get the patient transferred to ICU, and you can’t get a proper picture of the patient’s state until they’re already there in the ICU bed
26. Admissions due to a lack of beds in continuous surveillance unit
27. Poor appraisal of the situation (notably by telephone)
28. Unilateral decisions for surgery (e.g. a surgeon decides to operate on a patient who was refused for ICU care, and then once the patient is in theatre, the anesthesiologist calls the ICU because they can’t send the patient back to the ward because on noradrenaline…). Lack of expertise in the area among physicians in peripheral hospitals (nobody to discuss the prognosis of this or that disease with)
29. Lack of pluri-disciplinary discussions in advance
30. Need for a second opinion, from another ICU team, that also leans towards limitation of therapy
31. Patient’s advance directives were not known
32. Members of the medical team in disagreement amongst themselves
33. Patient referred by a peripheral hospital that is affiliated to our university hospital, but it’s hard to ascertain the reality of the situation. So the patient is de facto transferred “just in case”

**Domain 3: Possible CONSEQUENCES of a non-beneficial ICU stay**

Among 14 free-text responses under this domain, 7 (50%) correspond to items that were also in the list of proposed answers, and 7 (50%) provide new insights that could be relevant for future research.

The new insights include :

- The lack of available beds for a new admission could lead to a loss-of-opportunity for someone
- Caregivers could be prompted to resign or change to another unit
- Economic considerations, with consideration of the cost of unjustified ICU stays

1. No room for patients who need ICU
2. Therapeutic obstinacy, loss of motivation among the team – the impression that they’re wasting their time and life uselessly. Decline in the attractivity of ICU as a workplace.
3. Being unable to admit a patient who really needs ICU because beds are lacking
4. Burnout : days and nights spent wasting energy for a situation that you know is an impasse ; possible conflict with other professionals (who don’t understand futility) or even with families (who don’t understand the limitation even though they never knew what it was before, or who don’t understand the intensity of treatment in a situation whose seriousness they do understand)
5. Other ICUs saturated, no room for patients who really need ICU
6. Caregivers could run out of compassion
7. Exhaustion, lack of motivation, fed up, feeling that sometimes it’s a self-fulfilling prophecy, the physician specialist who prepared and anticipated nothing at all doesn’t have the charge of the patient at the end (because death is frequent in those cases), and doesn’t see the negative consequences, only retains the positive “in the end, I was right, they took my patient”
8. Caregivers quit the ICU because of that (ICM: ethics and intention to leave...), and we could do without that
9. Blocking beds for patients who need critical care
10. Monopolizing resources to the detriment of patients who need ICU
11. Cost
12. Risk of pathological grieving if the patient dies
13. Apathy, demotivation among the team
14. Difficulties withdrawing care

**Domain 4: MEASURES that could be implemented to avoid admissions that come to be deemed non-beneficial after the fact:**

Among the 10 free-text responses in this domain, 7 (70%) correspond to items that were already in the list of answers, either here, or in other domains (notably circumstances), and 3 responses (30%) provide new insights that could be relevant for future research.

The new insights include :

- Need for training in ethics for intensivists and non-ICU physicians
- Need to have an intensivist available 24/7 to make decisions in complex clinical situations and to counsel ICU and non-ICU physicians.

1. Improve training in ethics for all healthcare professionals
2. Give more information to patients and families about what ICU care is and its possible consequences
3. When a family or other physicians is putting « pressure » on you before admission: Re-discuss the situation as much as possible with the patient, the family or the medical team
4. More resources for the hospital
5. Involve the referring physicians more in the mangement during the ICU stay, and in dealing with the family and the caregiving teams (often quite angry) once the patient is in the ICU
6. Have medical files available 24/7. The problem stems more from a lack of access to information than from decisional problemes.
7. Standardized questionnaire for presenting the patient to the ICU physician (autonomy score, bedsores, advance directives, incurable disease, refusal of care, negligence, refused by other units,...)
8. Have medical files that are properly filled in and updated when you consult them in response to a request for admission
9. Have an outside opinion available 24/7, qualified in intensive care, to help physicians (ICU or not) with decisions about withholding or withdrawing therapy (second opinion)
10. Training of non-ICU physicians : identify patients at high risk of ICU for whom it might be justified to hold ethics conversations, help with the preparation of advance directives, help with ethics discussions….. Mobile team (including an ICU physicians and a nurse) for help with therapeutic decision-making. Presence of an intensivist at some multidisciplinary meetings.
